# Twenty Years of Advancements in a Radiotherapy Facility: Clinical Protocols, Technology, and Management

**DOI:** 10.3390/curroncol30070510

**Published:** 2023-07-22

**Authors:** Stefano Tomatis, Pietro Mancosu, Giacomo Reggiori, Francesca Lobefalo, Pasqualina Gallo, Nicola Lambri, Lucia Paganini, Francesco La Fauci, Andrea Bresolin, Sara Parabicoli, Marco Pelizzoli, Pierina Navarria, Ciro Franzese, Domenico Lenoci, Marta Scorsetti

**Affiliations:** 1 Medical Physics Service, Department of Radiotherapy and Radiosurgery, IRCCS Humanitas Research Hospital, Rozzano, 20089 Milan, Italy; 2 Department of Radiotherapy and Radiosurgery, IRCCS Humanitas Research Hospital, Rozzano, 20089 Milan, Italy; 3 Department of Biomedical Sciences, Humanitas University, Pieve Emanuele, 20090 Milan, Italy; 4 Development Strategic Initiatives Unit, IRCCS Humanitas Research Hospital, Rozzano, 20089 Milan, Italy

**Keywords:** radiotherapy, hypo fractionation, evolution, technology, optimization, throughput, management

## Abstract

Background: Hypo-fractionation can be an effective strategy to lower costs and save time, increasing patient access to advanced radiation therapy. To demonstrate this potential in practice within the context of temporal evolution, a twenty-year analysis of a representative radiation therapy facility from 2003 to 2022 was conducted. This analysis utilized comprehensive data to quantitatively evaluate the connections between advanced clinical protocols and technological improvements. The findings provide valuable insights to the management team, helping them ensure the delivery of high-quality treatments in a sustainable manner. Methods: Several parameters related to treatment technique, patient positioning, dose prescription, fractionation, equipment technology content, machine workload and throughput, therapy times and patients access counts were extracted from departmental database and analyzed on a yearly basis by means of linear regression. Results: Patients increased by 121 ± 6 new per year (NPY). Since 2010, the incidence of hypo-fractionation protocols grew thanks to increasing Linac technology. In seven years, both the average number of fractions and daily machine workload decreased by −0.84 ± 0.12 fractions/year and −1.61 ± 0.35 patients/year, respectively. The implementation of advanced dose delivery techniques, image guidance and high dose rate beams for high fraction doses, currently systematically used, has increased the complexity and reduced daily treatment throughput since 2010 from 40 to 32 patients per 8 h work shift (WS8). Thanks to hypo-fractionation, such an efficiency drop did not affect NPY, estimating 693 ± 28 NPY/WS8, regardless of the evaluation time. Each newly installed machine was shown to add 540 NPY, while absorbing 0.78 ± 0.04 WS8. The COVID-19 pandemic brought an overall reduction of 3.7% of patients and a reduction of 0.8 fractions/patient, to mitigate patient crowding in the department. Conclusions: The evolution of therapy protocols towards hypo-fractionation was supported by the use of proper technology. The characteristics of this process were quantified considering time progression and organizational aspects. This strategy optimized resources while enabling broader access to advanced radiation therapy. To truly value the benefit of hypo-fractionation, a reimbursement policy should focus on the patient rather than individual treatment fractionation.

## 1. Introduction

Radiation therapy (RT) is a therapeutic technique that employs high-energy radiation to target and destroy cancer cells, limiting their ability to grow and divide. It is a widely used treatment modality in oncology, offering localized treatment and often used in combination with surgery and chemotherapy for comprehensive cancer management. In particular, RT has experienced many clinical and technological advances in recent decades. This led to a growing interest of the RT community in the evaluation of objective data to endorse the crucial role and position of RT within the rapidly changing global oncology landscape [1,2,3].

On the clinical side, new hypo-fractionated clinical protocols were progressively introduced for different pathologies and body regions allowing access to effective and generally more advanced and comfortable therapies for a greater and greater number of patients [4,5,6,7,8,9,10,11,12,13,14,15,16,17,18,19,20,21].

Various measures and specifications can be used to evaluate machine performance, such as dose rate (the amount of radiation that can be delivered per unit of time), beam shaping (the shape, intensity, and modulation of the radiation beam), imaging capabilities, and patient positioning systems. To support the evolution of clinical protocols and improve radiation dose delivery, technological solutions were implemented, including fixed-beam intensity-modulated (IMRT) or, more recently, volumetric arc radiation therapy (VMAT) and flattening filter-free (FFF) high intensity beams. At the same time, new devices and methods were developed and applied to increase accuracy in patient positioning (e.g., image- and surface-guided radiotherapy, IGRT and SGRT, and six degrees of freedom couch, 6DOF) and to follow patient movements during delivery by means of tracking systems [22,23,24].

All these transformations led to an increased complexity of the radiotherapy workflow, and a consequent refinement of the software devoted to monitor the treatment quality, namely the record and verify systems (R&Vs). Initially developed to reduce the risk of treatment errors, R&Vs have evolved into complete RT information management tools, now called oncology information systems (OISs), integrating imaging systems, treatment planning computers, and treatment delivery systems. For each patient, several technical treatment-related parameters are generally automatically stored in the OIS database.

Once extracted, all this data can be organized in summary tables and charts, showing a general view of the department activities by relevant numbers. Data can also be exploited to develop suitable quality indicators, to demonstrate the existence of high quality operational standards or to apply some corrective actions, when necessary. Radiotherapy specific quality indicators, including technical features as the ones described above, have also been proposed as a recognized means for benchmarking on a national level [25].

Humanitas is a 750-bed university hospital, and a flagship hospital of the second largest private hospital group in Italy: the Humanitas Group. The Humanitas cancer center treats almost 7000 patients per year; the Hospital Radiation Therapy Department treats almost 3500 patients per year. Humanitas University, is dedicated only to life science subjects, where almost 50% of the students come from abroad (Europe, the USA, India, etc.). Our RT service started with two linear accelerators (linacs) in late 2003 growing up to the current number of five with updated technology progressively installed. All linac-based therapies in our department are managed by the same OIS (Varian Aria) storing a valuable amount of technical information in a single database.

The aim of this study is to provide a detailed overview of the evolution of our RT service over the past two decades, with a particular focus on the implementation of new hypo-fractionated clinical protocols and the utilization of state-of-the-art linear accelerator technology. By analyzing various parameters and metrics, this study aims to demonstrate the effectiveness of our RT service in delivering high-quality care to our patients. Moreover, the insights gained from this study can be useful for hospital management in quantifying and measuring the performance of the RT service and identifying areas for further improvement.

Since there are a lot of abbreviations throughout the paper, Table S1 is provided as Supplementary Material, with explanations and definitions of all the abbreviations.

## 2. Materials and Methods

Aria OIS was queried using different in house scripts. Such interrogations allowed us to extract several parameters related to treatment technique, fractionation, and equipment by structured queries involving multiple joined tables to obtain a full historical view of departmental activity. Main tables content ranged from a few million records for treatment data to several tens of millions for imaging-associated features. Other ancillary tables and views, including patient data were used to complete extraction.

A further elaboration was carried out to increase robustness and soundness of our data extraction. In fact, data were organized differently over the years with concomitant plans and linked treatments (such as sequential boost) often associated with different, manually created, therapy courses.

OIS software also evolved and expanded by changing the database structure. To manage this, we decided to consider related uninterrupted sessions of a patient in a treatment series as belonging to the same therapy course. Other data processing was performed to filter non-relevant records, such as QA or training sessions not attributable to real patient irradiations.

Despite the inclusion of certain tools in the oncology information system to facilitate detailed data reporting, such as patient counts and associated imaging information, the available software packages lacked the required level of flexibility to efficiently extract and manage the vast database accumulated over a period of 20 years. To enhance support for customized data filtration, aggregate treatment records, and therapy session management, we deemed low-level SQL programming to be a more suitable choice [26].

For the analysis, we chose a collection of parameters we could readily relate to the main aspects of the therapy course (Table 1)

Other parameters of interest to describe department’s activity were further derived from the queries and defined as follows on a yearly basis: linacs, i.e., the number of linear accelerators; new patients per year (NPY), i.e., the number of patients starting at least one therapy course in a specific year; daily patient workload (DPW): total number of patients treated in the department in a day; and daily patient linac workload (DPLW), defined as DPW divided by linacs.

The average values of daily workloads (DPW, DPLW), number of fractions (NOF, Table 1), linacs, and WS8 (Table 1) were evaluated as representative figures in a year to account for their variation over time. Machine throughput was also calculated, defined as DPW/WS8.

To follow the evolution in our center of linacs characteristics over time, we propose a technology score (TS) based on essential features, covering different kinds and performances of dose delivery techniques, radiation beams, ability to perform IGRT, and online verification of patient positioning summarized in the following categories:

(1) Standard Delivery: 3D CRT and Portal Imaging IGRT
(2) Advanced Delivery: IMRT-VMAT irradiation
(3) CBCT IGRT: Cone beam CT IGRT capability
(4) FFF: Flattening Filter Free Beams capability
(5) 6DOF: Six Degrees of Freedom Couch
(6) SGRT: Surface Guided Radiotherapy System

The associated TS is assigned to each linear accelerator by simply adding one point for each of the previous items from one to six. Averaged TS in our department is also calculated and added to our descriptive statistics on a yearly basis. Standard linear regression was performed to quantitatively describe both annual trends and relationships between selected variables. Restricted or piecewise analyses were applied, when appropriate, for significant trends, excluding outliers. Standard descriptive statistics and Mann–Whitney test were used for nonparametric evaluation and test of data between groups. Any statistical estimation was considered “significant” for corresponding *p*-values less than 0.05, and “highly significant” for *p*-values below 0.01. Statistical computations were performed with the STATA statistical software package (StataCorp. 2017, Stata Statistical Software: Release 15. College Station, TX, USA: StataCorp LLC).

## 3. Results

Table 2 shows history and main features of linac installations in our department, according to commissioning and decommissioning times. Technology scores are also included in the table.

**Figure 1:**
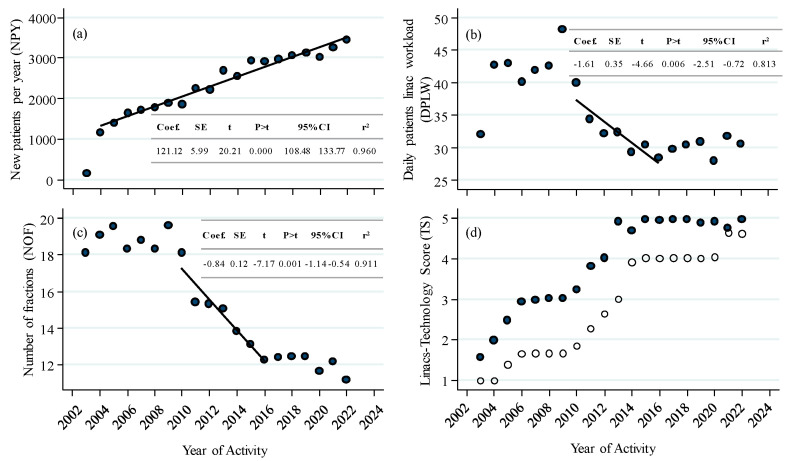
Evolution of a radiotherapy facility: number of new patients per year or daily treated per linac (**a**,**b**); (**c**) average fractionation. Regression lines plotted for significant trends. Plots inserts, regression details; (**d**) number of linac installations (closed symbols) and associated average technology score (TS, open symbols).

Figure 1 shows the yearly trend of new patients treated (NPY), daily patient linac workload (DPLW), number of fractions (NOF), linacs, and technology score (TS) since the beginning of the departmental activity in November 2003.

**Figure 2:**
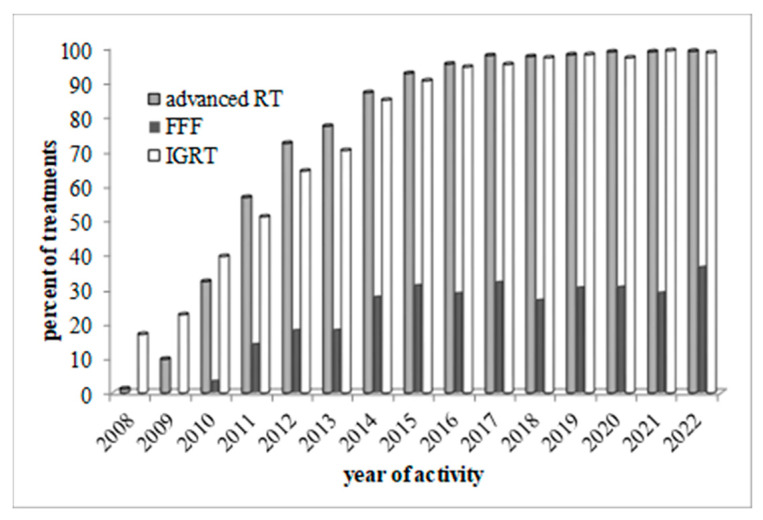
Incidence over time of the use of advanced techniques (advanced RT, gray), flattening filter-free beams (FFF, dark gray) and image-guided radiotherapy (IGRT, white).

Incidence in the use of advanced (IMRT or VMAT) techniques, FFF beams and IGRT protocols in therapy courses is shown in Figure 2 as a function of time since 2008.

**Figure 3:**
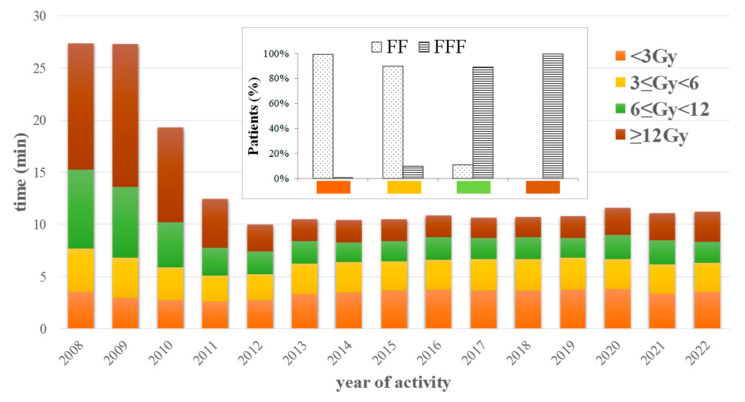
Mean treatment time plotted per year and different fraction dose categories according to legend’s colors. The use of flattened (FF) vs. flattened filter-free (FFF) beams is represented in the figure insert according to the same dose categories for year 2022.

Mean treatment time year by year as extracted from machine records is plotted in Figure 3, according to four different dose prescription categories. Different use of flattened vs. flattened filter-free beams is shown in the figure insert according to the same dose categories.

**Figure 4:**
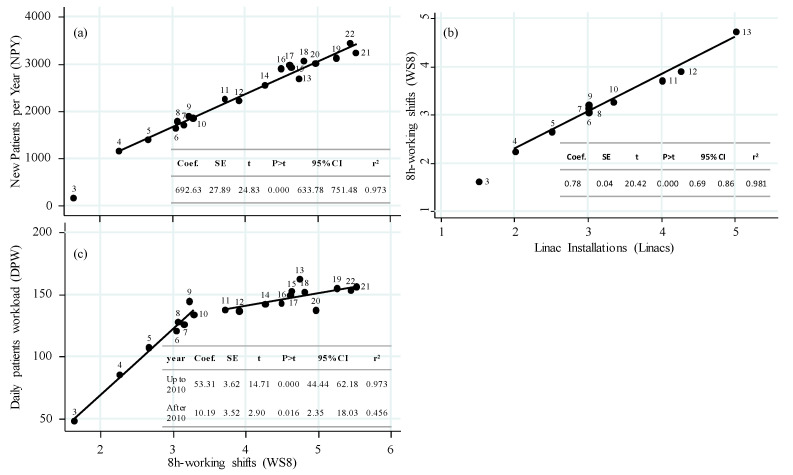
Ability of working shifts to manage total patients burden both new per year (**a**) and on-treatment daily (**c**); (**b**) ability of linacs to absorb working shifts. Labels inside plots refer to the year of evaluation. Inserts give details of plotted regressions lines.

Ability to manage patients, both new per year and daily treated by means of allocated working shifts at treatment bunkers is shown in Figure 4. Capability of each added therapy machine to “absorb” working shifts at the bunkers is also provided in the Figure 4.

Machine throughput up to and after 2010 was evaluated as 39.9 [38.5–41.0] and 31.9 [28.8–33.6] patients per WS8, respectively, (median [iqr]) *p* = 0.003.

## 4. Discussion

This study offers an insight into the activity and evolution of an RT center over two decades. Although monocentric, to the best of our knowledge, this is the first study able to describe this evolution based on extensive data mining in such a wide time interval. In spite of the widespread use of quality indicators, both for technical and clinical aspects of the radiotherapy workflow, data collection and availability of a complete information set for all patients and treatments may be limited due to the applied methodology or study purposes, especially for national multi-center surveys [25].

Therefore, we believe that systematic data collection, even if it is carried out in a single albeit representative institution, can be useful as a starting point, or as an example to follow, regarding the adopted methods. These approaches utilize direct querying of technical attributes that are typically automatically stored by the system, making them more suitable for a detailed analysis compared to manually sampled data input. In order to enhance patient care and improve research quality, it is important to foster improved connectivity between databases of various providers and radiotherapy centers in the future [27].

After activity started in late 2003, new patients treated in our facility show an overall increasing trend resulting in 121 additional patients per year (Figure 1a). In contrast, DPLW shows a significant drop, markedly between 2010 and 2016 (Figure 1b), quantified in −1.6 patients daily treated per year on a single accelerator. Since the machine workload can be simply modeled as DPLW = (NPY × NOF)/(WD × linacs), where WD are the working days in a year, the observed drop in NOF (Figure 1c) paired with the increase in linacs (Figure 1d) can easily explain the observed DPLW fall, despite the number of new patients increasing.

Also, a fast rise in technology content is observed since 2010, with TS ranging from <2 up to about 4 in 2014, and, finally, above 4.5 after the installation of linac 8 in early 2021 (Figure 1d; Table 2).

The first “new generation” machines were introduced in our department in 2010, equipped with advanced delivery, CBCT IGRT and FFF beams all together and with a TS ≥ 4 (Table 2). These features allow us to treat patients with improved positioning control and more efficient delivery techniques, suitable to better spare critical structures close to the target compared to traditional accelerators and machines with a TS below three. Moreover, such advanced linac models feature fast dose rates, up to four times the traditional ones, sparing treatment times, especially when high fractional doses (≥12 Gy) are involved.

To the best of our knowledge, despite their widespread use and the need of a systematic classification of structural quality indicators in radiotherapy [25], there is currently no specific score available or reported in the guidelines for performing a quantitative assessment of the technology content of linear accelerators. Given the lack of approved definitions of a “Technology Score” from organizations such as IAEA or AAPM, our TS metric stands out as original. We consider it a valuable tool for describing and comparing technological advancements, albeit at a fundamental descriptive level. Nevertheless, taking into account our specific situation and history as described in Table 2, this index remains valid, at least on an ordinal scale, as it incorporates previous TS levels. The TS improved with each new installation or substitution of an older linac. The history of linac 5 is particularly noteworthy. It was installed in 2012 as a replacement for a single energy machine (linac 1) without IMRT/VMAT capability and TS = 1. However, at that time, the TS level (two) of linac 5 was worse than that of one of the latest installations (linac 3 and 4, TS = 3 and 4, respectively). The machine was replaced after only eight years, in 2020, with a linac that had a TS level of five. Therefore, we emphasize that machine substitutions should be performed using state-of-the-art technology; otherwise, they may become obsolete rather quickly, resulting in unnecessary resource displacement.

IGRT application over time was found to grow and follow the trend of advanced therapies up to a systematic implementation in recent years (Figure 2). FFF beams were also used (Figure 2) to better manage high fraction doses by reducing treatment times. The close relation between treatment times and FFF adoption as shown in Figure 3 is clear.

Since 2010, the introduction and increasing use of new technologies have allowed for the implementation of hypo-fractionation protocols within specific Phase 2 studies [28,29,30]. These protocols have led to a reduction in the number of fractions from about 18 to 12 within a span of seven years, as shown in Figure 1c. The reduction occurred at a rate of −0.8 fractions per year.

As treatments have become more complex, there has been a decrease in machine throughput, from 40 to 32 patients per 8 h shift. This drop in capacity is due to the need for more resources and personnel to manage the more sophisticated radiotherapy processes while ensuring proper quality [31,32]. The increase in initial treatment sessions and the utilization of adaptive protocols, at least for certain body regions, may also contribute to complexity rise [33]. The dependence of daily treatments on allocated time has two distinct slopes with a breaking point in 2010, which limits the gain in daily capacity from 53.3 to 10.2 patients when adding a work shift (Figure 4c). Although radiation therapy may seem potentially more expensive, it is possible to provide better and more advanced therapies while also addressing the increasing costs associated with the implementation of enhanced technologies that have been shown in these years to involve more time for the treatment session.

In fact, in terms of new patients, there has been a steady growth observed. The regression analysis shows that for each added WS8, there is a good correlation (r^2^ = 0.973, *p* = 0.000), assigning 692.6 NPY, regardless of the evaluation period (Figure 4a). This management, optimized thanks to hypo-fractionation, benefits both patients and the healthcare system. If the reimbursement policy focuses more on patients rather than individual treatment fractions, this trend provides better sustainability chances for the hospital and promotes cost-saving policies, as recommended by other studies [34,35,36,37].

The studies aim to compare the costs of delivering radiotherapy treatments for different body regions using conventional and hypo-fractionated regimens also considering cases where access to radiotherapy resources is limited. The authors provide estimates based on different cost calculation models and cancer incidence public data. By these projections, they conclude that hypo-fractionation offers a substantial economic benefit, is less burdensome to patients, and provides a cost-efficient mechanism to treat an increasing number of patients within the existing capacity. It is worth noting that reducing the duration of the treatment with a therapy shorter schedule may result in reduced toxicities, also and above all allowing an expansion of the possible clinical indications of radiation treatments with a consequent increase in survivals.

All the examined considerations and conclusions from the literature anticipate and match real-world results like the ones presented in this paper. In addition, based on our 20-years’ experience, we show a detailed view of the timing required for implementing new protocols in a seven-year process bringing a contextual drop in machine workload, also allowed thanks to the equipment growth (Figure 1).

Measurements of the relationships occurring between technology or clinical innovation and important organization characteristics such as department capacity, machine occupancy, throughput and dedicated resources are generally limited in current published studies due to the sampling and short evaluation time. Nevertheless, they validate the importance of ongoing monitoring of these variables by offering examples of organizational changes resulting from observational analyses [38,39].

Of course, although representative, our results are partial because they are related to a single institution, and further improvements are necessary to extend the validity of our findings that could not hold the same in other centers or contexts.

Work is in progress to include other hospitals of our network in the analysis, considering different situations in order to obtain a better degree of generalization in a multi-center study. At the same time, we need to consider additional information and details about the case mix of our patients. Significant differences in the composition of pathologies, body sites, or staging may impact the possibility to generalize our conclusions.

To overcome this problem and allowing a comparison among different centers all over the world, the definition of a hypo-fractionability coefficient may be of use by assigning higher weights to specific clinical situations where more chances of reducing fractions can be attributed according to the accepted guidelines.

In general, the reported clinical applications of therapy course fractionation in our department have the overall impact of enhancing access to radiotherapy, thereby aligning with the recommendations of the Lancet Oncology commission. According to this and other studies [3,40], there is compelling evidence that investment in radiotherapy not only enables treatment of a large numbers of cancer cases to save lives, but also brings positive economic benefits.

It is difficult to estimate how fractionation schemes will evolve in the future. Our tendency is to treat patients in shorter times when this possibility is supported by clinical evidence. Although current clinical research is rapidly changing the evidence-base for fractionation, supporting the feasibility of reduced courses, some limitations still hold related to the specific radiosensitivities of some tissues at risk (e.g., for H&N treatments) or to the extension of the irradiated volume, especially for extreme hypo-fractionation protocols. To allow further steps towards a drastic reduction in the number of fractions, a proper use of particle therapy, e.g., protons, and new applications exploiting radiobiological research findings, such as flash and/or grid therapy, could play a main role in the coming years [41,42].

In 2020, there was a decrease in the number of patients treated (117 patients, 3.7%, Figure 1a) and machine occupancy (3 patients, Figure 1b). Owing to a stable machine layout since 2015 (Figure 1d), the observed decline is to be attributed to the COVID-19 pandemic and patients’ constrained access to healthcare facilities imposed by national regulations. A fraction mitigation policy, specifically implemented in 2020 to avoid patient crowding according to international guidelines [43], can explain the observed 0.79 NOF reduction (Figure 1c). However, all parameters have since recovered to pre-COVID levels in 2021 (Figure 1a–c).

The data mining methods described in this paper demonstrate their usefulness in tracking various features stored in the departmental OIS. Our results and methods can be further extended and utilized by others to plan, manage, and monitor the necessary technology and clinical changes, thus raising the awareness of management staff on the associated impact, timing, benefits, and costs.

## 5. Conclusions

In conclusion, the data presented in this study illustrate the technological advancements that have occurred in radiotherapy over the past two decades to facilitate the implementation of hypo-fractionated clinical protocols and improve patient access to advanced treatments. Hypo-fractionation has played a crucial role in driving the evolution of technology while ensuring the optimal utilization of resources. Although the COVID-19 pandemic caused a reduction in patient treatments, the implementation of a safer patient management policy, which included a reduction in fractionation, helped prevent patient overcrowding. Despite this setback, our department was able to return to pre-pandemic activity levels in 2021. These findings provide valuable information for clinical and administrative management teams to plan, implement, and monitor necessary improvements, ensuring the continued integration of technological advancements with the evolution of clinical protocols.

As an essential step in the large-scale adoption of hypo-fractionation beyond the COVID-19 pandemic context, it is crucial to prioritize a reimbursement policy that focuses on the patient rather than individual treatment fractionation, in order to be able to truly value the benefit of hypo-fractionation.

## Figures and Tables

**Table 1:** Technical and clinical data extracted from our departmental OIS.

Parameter	Specification
Date	session date
Linac	treatment machine (linear accelerator)
WS8	mean total operational hours per day of each linac normalized by 8 h (i.e., the standard working shift)
Treatment time (TRT)	total treatment irradiation time
Dose	prescribed treatment dose (Gy)
Number of fractions (NOF)	total number of fractions in a therapy course ^1^
Technique	standard (3D CRT-rotational arc) or advanced (IMRT-VMAT) therapy
FFF	use of high intensity flattening filter free (FFF) beams
IGRT	use of Image Guided RT in a therapy session

^1^ Fractions summed for sequential treatments, merged for multiple concomitant targets.

**Table 2:** Main features of the linacs installed in our department.

Machine	Replacing ^1^	Operation (Start–End)	Main Features	TS ^2^
Linac 1	(new)	November 2003–December 2011	Standard Delivery	1
Linac 2	(new)	November 2003–December 2013	Standard Delivery	1
Linac 3	(new)	July 2005	Standard Delivery, Advanced Delivery, CBCT IGRT ^3^	3
Linac 4	(new)	September 2010	Standard Delivery, Advanced Delivery, CBCT IGRT, FFF	4
Linac 5	Linac 1	January 2012–November 2020	Standard Delivery, Advanced Delivery	2
Linac 6	(new)	October 2012	Standard Delivery, Advanced Delivery, CBCT IGRT, FFF, 6DOF	5
Linac 7	Linac 2	March 2014	Standard Delivery, Advanced Delivery, CBCT IGRT, FFF, 6DOF, SGRT	6
Linac 8	Linac 5	February 2021	Standard Delivery, Advanced Delivery, CBCT IGRT, FFF, 6DOF	5

^1^ Replaced therapy machine, ^2^ technology score, ^3^ available since 2008.

## Data Availability

The data presented in this study is available on request from the corresponding author.

## Supplementary Materials

The following supporting information can be downloaded at: https://www.mdpi.com/article/10.3390/curroncol30070510/s1, Table S1: List of abbreviations.
