# Supplementary material for: Twenty Years of Advancements in a Radiotherapy Facility: Clinical Protocols, Technology, and Management
Source: Curr Oncol. 2023 Jul 22;30(7):7031–42. doi: 10.3390/curroncol30070510 (PMC10378035; doi:10.3390/curroncol30070510)
Supplement: Supplementary file 1 [file curroncol-30-00510-s001.zip › curroncol-2419484-supplementary.pdf]

Current Oncology

Supplementary Material

Table S1. List of abbreviations

Abbreviation	Definition	Explanation
**3D CRT**	3D conformal radiotherapy	radiotherapy technique using static or rotating beams without beam modulation, unable to provide non convex target conformality
**6DOF**	Six degrees of Freedom Couch	positioning device used in radiotherapy that allows the patient to be moved in six different directions translation for x, y, and z axes, and rotations: couch table, pitch and roll movements
**CBCT**	Cone-Beam Computed Tomography	imaging technique used in radiotherapy to obtain tomographic 3D images with a cone-shaped X-ray beam
**DPW**	Daily Patient Workload	number of patients treated on a daily basis in the department
**DPLW**	Daily Patient Linac Workload	number of patients treated on a daily basis per single linac
**FF**	Flattening Filter	component of the linear accelerator used in radiotherapy that helps to evenly distribute the radiation beam
**FFF**	Flattening Filter Free	radiotherapy technique that eliminates the use of the flattening filter in the linear accelerator
**Gy**	Gray	unit used to measure the absorbed dose of radiation. It represents the amount of energy (in joules) deposited per unit mass (in kilograms) of tissue
**IGRT**	Image Guided Radiotherapy	radiotherapy technique that utilizes imaging technology, such as CBCT, to accurately guide and adjust the radiation treatment based on the patient's anatomy and tumor position
**IMRT**	Intensity Modulated Radiation Therapy	technique developed to deliver radiation doses with varying intensity levels achieved by modulating of the radiation beam
**NOF**	Number of fractions	total number of fractions in a therapy course
**NPY**	New Patient per Year	number of newly diagnosed patients who begin receiving radiotherapy treatment in a given year
**OIS**	Oncology Information System	specialized software system used in oncology departments to manage and organize patient information, treatment planning, scheduling, and other aspects of cancer care. It is an evolution of a R&V system
**QA**	Quality Assurance	procedures implemented to ensure that the radiotherapy equipment, treatment plans, and delivery are accurate and adhere to established standards and protocols.
**R&V**	Record and Verify System	specialized software that captures, stores, and verifies critical information related to radiation treatment. It records details such as patient data, treatment plans, machine settings, and actual treatment delivery parameters
**RT**	Radiotherapy	medical treatment that utilizes high-energy radiation, such as X-rays or gamma rays, to target and destroy cancer cells or shrink tumors

**SGRT**	Surface Guided Radiotherapy	Technique using real-time monitoring of the patient's body surface to ensure accurate radiation delivery in radiotherapy
**TS**	Technology Score	metric designed to characterize the technological advancement of a linear accelerator
**VMAT**	Volumetric Arc Radiotherapy	radiotherapy technique that uses a rotating gantry to deliver radiation in a continuous arc while dynamically modulating its intensity
**WD**	Working Days	days in a year available for work, excluding weekends and public holidays
**WS8**	8-hours Work Shift	standard full-time work period of 8 consecutive hours
